# Dual intra-articular injections of corticosteroid and hyaluronic acid versus single corticosteroid injection for ankle osteoarthritis: a randomized comparative trial

**DOI:** 10.1186/s12891-025-08488-0

**Published:** 2025-03-11

**Authors:** Inha Woo, Jeong-Jin Park, Chul Hyun Park

**Affiliations:** 1 The Armed Forces Daejeon Hospital, Daejeon, Republic of Korea; 2 Korea Armed Forces Athletic Corps, Mungyeong, Gyeongsangbuk-do Province Republic of Korea; 3 Department of Orthopaedic Surgery, College of Medicine, Yeungnam University, 170 Hyeonchung-ro, Nam-gu, Daegu, 42415 Republic of Korea

**Keywords:** Ankle, Osteoarthritis, Injection, Steroid, Hyaluronic acid

## Abstract

**Background:**

Intra-articular corticosteroid injection is commonly used for pain relief in ankle osteoarthritis (OA). The effects of corticosteroids (CS) are short-lived, whereas hyaluronic acid (HA) have longer-lasting effects. The objective was to compare the efficacy of dual injections of CS and HA to CS alone. We hypothesized that intra-articular injections of dual agents would be more effective than CS alone.

**Methods:**

A single-blind, randomized, controlled trial was designed to investigate this hypothesis. 135 patients with ankle OA were enrolled into an intra-articular CS injection group (CS group, *n* = 61) or dual HA plus CS injection group (CS + HA group, *n* = 74). The CS group received 1 mL of corticosteroid and 1 mL of 0.5% bupivacaine and 1 mL of normal saline once, and the CS + HA group received 3 mL of a total of 5 mL mixtures containing 2 mL of HA, or 1 mL of corticosteroid, 0.5% bupivacaine, and normal saline in the first week, followed by 2 mL of HA in the second and third weeks. Clinical evaluations were performed before injection, 6 and 12 weeks after the first injections. The Ankle Osteoarthritis Scale (AOS) was used as the primary outcome measure, and the Visual Analogue Scale (VAS), Short Form Health Survey (SF-36), and complications were used as secondary outcomes.

**Results:**

The mean AOS change from baseline was significantly greater in the CS + HA group than in the CS group at 6 (*p* ≤ 0.01) and 12 weeks (*p* ≤ 0.01). The mean VAS change from baseline was significantly greater in the CS group than in the CS + HA group at 6 weeks (*p* = 0.023), but not at 12 weeks (*p* = 0.731). The mean SF-36 change from baseline was not significant between the CS and CS + HA groups at 6 (*p* = 0.416) and 12 weeks (*p* = 0.215).

**Conclusions:**

The combination of corticosteroid and HA injection is more effective than corticosteroid alone in relieving pain in ankle OA.

**Trial registration:**

Clinical Research Information Service in South Korea, KCT0008690 // Registration Date (First Posted): July 21th, 2023 (http://cris.nih.go.kr).

**Supplementary Information:**

The online version contains supplementary material available at 10.1186/s12891-025-08488-0.

## Background

### Introduction

Ankle osteoarthritis (OA) can have a devastating impact on quality of life due to pain and functional limitations [1–4]. In addition, unlike hip or knee OA, ankle OA affects a large proportion of younger age groups due to its high relevance of post-traumatic etiology [1]. Owing to its onset at a relatively young age, appropriate nonoperative treatments including lifestyle modification, analgesics, orthotics, and intra-articular injections are critical to delay operative treatment [4]. Based on the findings of Tejero et al. [1] intra-articular injections demonstrated relatively better outcomes compared to other conservative treatments like other orthotics or braces for ankle OA. This supports the rationale for focusing on injection therapies in this study.

One of these options, intra-articular corticosteroid injection was first introduced as a treatment for OA in 1951 and performed worldwide [5]. Although there is a paucity of published data regarding the influence of an intra-articular corticosteroid (CS) injection for ankle OA, some studies have shown its improvements of clinical symptoms for ankle OA [5–7]. Its mechanism of action is to decrease inflammation in synovial tissues and inflammatory cell numbers in affected joints [8]. Currently, intra-articular CS injection is a widely adopted non-surgical treatment for managing OA in many joints due to rapid action and cost-effectiveness [9]. However, intra-articular injection of CS is effective for only up to 4 weeks, and long-term corticosteroid treatment can cause joint destruction and tissue atrophy [10, 11].

Hyaluronic acid (HA) which was first approved by the Food and Drug Administration (FDA) in 1997 for knee OA, has been administered in various joints, and shown to be effective in OA as a visco-supplementation [12, 13], which refers to synovial fluid replacement by intra-articular injection [14, 15]. HA, which consists of normal joint fluid, is a high-molecular polysaccharide composed of N-acetylglucosamine and glucuronic acid [16, 17]. Intra-articular injection of HA replenishes joint fluid loss, protects damaged articular cartilage, and eventually diminishes pain by relieving OA-related inflammatory changes [18]. Previous randomized controlled trials showed that intra-articular HA injection has long-term effects similar to those of intra-articular corticosteroid injection and fewer side effects [17, 19–21].

Generally, the combined use of CS with various agents, including local anesthetics, has been well-documented in numerous studies. The design of this present study was significantly affected by such prior research [22–25]. However, to our knowledge, no report has been issued on the effect of dual injection of CS and HA for ankle OA. Thus, the purpose of this study is to prospectively show that dual therapy offers a superior alternative for both short- and long-term management. Given the cost-effectiveness and the insurance regulations of various nations including South Korea, CS injection is widely used clinically. We hypothesized that dual intra-articular injections of HA and CS would provide faster, longer-term pain relief than corticosteroid alone in patients with ankle OA.

## Materials and methods

### Study design

This study was approved by the institutional review board and was conducted following approval of the health authority by Clinical Research Information Service (CRIS) of Korea Disease Control and Prevention Agency (KDCA). The trial has been registered in the CRIS.nih.go.kr database. This also adheres to the CONSORT 2010 guidelines and was performed in accordance with the described procedures in the approved study protocol. Informed consent was obtained from each patient. There have been no changes in the trial protocol after the trial commencement. The detailed protocol was provided in Supplementary Information.

### Participants

The study was conducted on patients that met the inclusion and exclusion criteria in full as described in Table 1. This study was conducted to evaluate the effect of adding HA injection to CS injection. Therefore, the control group was set as CS injection alone, and the experimental group was set as dual injection of CS and HA. The injection method of each agent was determined based on the method implemented in previous studies [23, 26, 27]. The CS injection was performed once, and the HA injection was performed three times at one-week intervals [22]. This study is not a fully single-blinded because of the different number of injections between the two groups, however, participants were not informed about the number of injections to eliminate bias that might be introduced by the different number of injections.

**Table 1:** Inclusion and exclusion criteria

Inclusion criteria
Adult patients (> 18 years) who failed to respond to ≥ 3 months of other conservative treatments
Primary ankle osteoarthritis
Varus ankle osteoarthritis
Patients with a follow-up period of > 12 weeks
**Exclusion criteria**
Rheumatoid arthritis or osteonecrosis caused by another illness (hemophilia or Charcot arthropathy)
Valgus ankle osteoarthritis
Traumatic ankle osteoarthritis
History of intra-articular injection or surgery related to ankle osteoarthritis
Suspicion of pyogenic or inflammatory arthritis
Recent infection history or current cellulitis
Other confounding conditions, such as severe vascular insufficiency and recent sciatica

Patients were randomized to receive an intra-articular CS injection (CS group) or intra-articular injections of CS and HA (CS + HA group). Patients were followed-up for 6 and 12 weeks after first injections. Only unilateral injections with more severe side were performed and counted in this study. In this study, we defined traumatic arthritis as having a fracture around the ankle, surgery for severe ankle instability, or three or more repetitive sprains, and excluded these cases from the study.

### Randomization

All the study was designed parallelly. From November 2015 to July 2022, patients who were decided to receive the intra-articular injection due to ankle OA via our tertiary orthopedic hospital were recruited and randomly assigned to the CS or CS + HA group by using a permuted block design of two. Randomization was conducted using a computer-generated allocation program (nQquery Advisor PPS 6.01, Saugus, MA, USA) that assigned numbers in strict chronologic. Randomization was stratified by age and OA stage, as defined by the modified Takakura classification [28]. The randomization was conducted by an independent researcher. Each study participant was allocated a unique randomized number.

### Injection methods

Before first injections, radiographic evaluations were performed using weight-bearing ankle anteroposterior (AP) and lateral radiographs and hindfoot alignment radiographs [29, 30]. All radiographs were obtained digitally, and radiographic parameters were measured using a Picture Archiving Communication System (PACS; Infinity, Seoul, Korea). Ankle OA was classified using the modified Takakura classification. An orthopaedic attending professor and an orthopaedic resident independently determined classifications twice at four-week intervals independently. When disagreement arose, the patient’s radiograph was replaced with another considered more representative until consensus was achieved.

A standard sterile skin preparation technique was performed around the ankle joint, and an intra-articular injection was performed medial to the tibialis anterior tendon in the same location used for the anteromedial portal during ankle joint arthroscopy. Since this study was undertaken to investigate the effects of HA plus CS versus CS alone, we decided to add HA to the conventional intra-articular corticosteroid injection regimen [31]. In the CS group, 3 mL of mixture including 1 mL of corticosteroid (2.5 mg/mL, Triam^®^, ShinPoong Pharmaceuticals, Seoul, Korea), 1 mL of 0.5% bupivacaine (bupivacaine HCl^®^, Hana Pharm, Seoul, Korea), and 1 mL of normal saline were injected slowly [32]. In the CS + HA group, 2 mL of HA (sodium HA, molecular weight, 3000 kDa; 2 mL, Hyruan Plus^®^; LG Life Sciences, Iksan, Korea) and 3 mL of mixture including 1 mL of corticosteroid, 1 mL of 0.5% bupivacaine, and 1 mL of normal saline were mixed and 3 mL of total 5 mL was injected on the first week, followed by single injections of 2 mL of HA on the second and third weeks [26, 27]. Patients taking analgesics or NSAIDs stopped at least 7 days before the pre-injection assessment. All oral analgesics were prohibited during the study, and patients who required additional analgesics for uncontrolled pain were excluded from the study.

### Outcome assessments

The clinical evaluation was performed by an experienced nurse blinded to group-allocation. Clinical evaluations were performed prior to injections (baseline) and at 6 and 12 weeks after first injections. Clinical evaluations were performed using the Ankle Osteoarthritis Scale (AOS), the 36-item Short Form health survey (SF-36), the visual analogue scale (VAS), and complications after injection [33, 34].

AOS is a patient-rated, validated measurement that contains pain and disability subscales (9 items each). Each item ranges from a score of 0 representing no pain or disability to a score of 10 indicating worst pain or disability [33, 35]. VAS for pain is commonly used, and a score of 0 represents ‘no pain’ and a score of 10 ‘worst pain imaginable’. SF-36 is one of the most widely used generic scales and has been tested for validity and reliability. It includes eight scaled scores, which are the weighted sums of the questions in their respective sections. These eight scales are further aggregated into two summary measures: the Physical Component Summary (PCS) and the Mental Component Summary (MCS). PCS score ranges from 0 to 100, standardized with a mean of 50 and a standard deviation of 10 in the general US population [36, 37].

AOS was used as the primary outcome assessment tool due to its reliability and specificity for evaluating ankle OA [33, 34]. Secondary outcomes included VAS, SF-36, and complications after injection. All the parameters before and after injection were evaluated within the CS and CS + HA groups, and between these groups. Given the significant baseline differences in VAS and SF-36 scores, median changes from baseline to 6 and 12 weeks after injection were compared, along the occurrence of complications.

In addition, subgroup analysis was performed to analyze clinical outcomes according to staging of ankle OA in the CS and CS + HA groups. AOS, SF-36, and VAS before and after injection were analyzed in each stage of ankle OA by modified Takakura classification [28, 29] in the CS and CS + HA groups.

Patients were sufficiently informed of possible complications. For assessment purposes, complications were dichotomized as major or minor. Minor complications included injection site pain and superficial swelling manageable without special procedures, and major complications included neurovascular injury and deep ankle joint infection requiring additional treatment. Complications were assessed and recorded at each follow-up visit. The discontinuation of the study protocol was set as the occurrence of major complications or voluntary subject’s desire.

### Sample size

To determine the appropriate number of participants, we performed a power analysis. This helps ensure that our study is statistically reliable. We used a two-tailed matched-pairs t-test with the following settings: an effect size of 0.25 for our primary outcome measure, a significance level (alpha) of 0.05, and a power of 0.80. Additionally, we factored in a 10% dropout rate [38, 39]. Based on these criteria, we calculated that we needed a total of 141 patients to achieve reliable results.

### Statistical analysis

We analyzed the data using SPSS 17.0 software (SPSS, Chicago, IL). Results are presented as means and standard deviations. First, we checked if the data followed a normal distribution using the Shapiro-Wilk test. For comparisons between groups, we used chi-squared tests for categorical data and independent t-tests for continuous data. Within each group, we compared pre- and post-injection data using paired t-tests. We also used Analysis of Variance (ANOVA) for within-group comparisons over time, applying a Bonferroni post hoc test for pairwise comparisons. (small 0.01; medium 0.06; large 0.14). Effect sizes were reported using partial eta squared values. A significance level of *p* < 0.05 was used, adjusted to 0.016 for repeated measures ANOVA to account for multiple comparisons.

### Minimum clinically important difference (MCID)

The MCID represents the smallest change in a treatment outcome that an individual patient would identify as important [40]. To calculate MCID, we used two main approaches: anchor-based and distribution-based methods [41]. The anchor-based method relies on external criteria or ‘anchors,’ such as patient-reported improvement [42, 43]. The distribution-based method uses statistical calculations, often defining MCID as half of the standard deviation of the change scores between pre- and post-treatment [44]. While there is no universally superior method, these approaches help us understand the clinical significance of our results.

## Results

### Patient characteristics

**Fig. 1:**
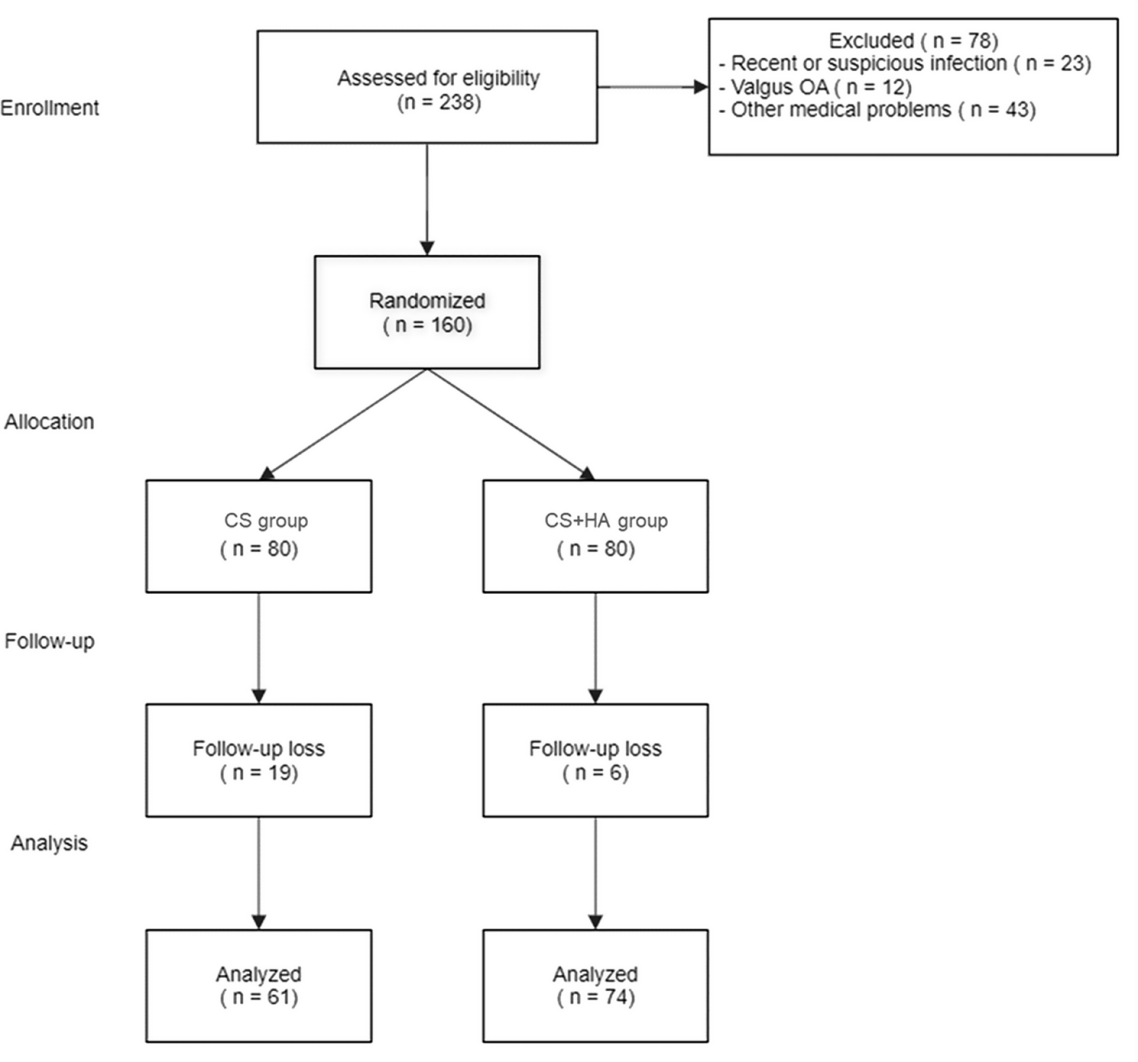
Flow diagram of patient enrollment

From November 2015 to July 2022, 238 patients with ankle OA were screened. After exclusions (Figs. 1), 135 patients met the inclusion criteria and were randomly assigned to either the CS group or the CS + HA group. The demographic details of these groups are summarized in Table 2, showing no significant differences between them.

**Table 2:** Demographic data

	CS group (*n* = 61)	CS + HA group (*n* = 74)	*p*-value
Age (years)*	60.5 ± 9.46 (29–82)	58.8 ± 9.84 (38–86)	0.319
Sex (male / female)	39 (60.9%) / 22	43 (58.1%) / 31	0.490
BMI (kg/m^2^)*	25.7 ± 3.0	25.5 ± 3.6	0.698
Modified Takakura classification			0.942
2	35 (57.4%)	45 (62.2%)	
3A	14 (23.0%)	15 (20.3%)	
3B	8 (13.1%)	8 (10.8%)	
4	4 (6.6%)	5 (6.8%)	

BMI, body mass index

*The continuous values are presented as means ± standard deviations with ranges and categorical values as numbers and percentages

### Primary outcome

For the Ankle Osteoarthritis Scale (AOS), there was no significant improvement in the CS group at 6 (*p* = 0.761) and 12 weeks (*p* = 0.893) after injection compared to baseline. However, the CS + HA group showed significant improvements at both 6 (*p* ≤ 0.01) and 12 weeks (*p* ≤ 0.01) compared to baseline. Overall, the improvement in AOS scores was significantly greater in the CS + HA group than in the CS group (repeated measures ANOVA *p* < 0.001), with mean changes from baseline being significantly higher in the CS + HA group at both 6 (*p* ≤ 0.01) and 12 weeks (*p* ≤ 0.01). These results were depicted in Table 3.

**Table 3:** Mean changes between baseline versus 6 and 12 weeks after injection

	Outcomes (95% CI)	CS group	CS + HA group	*p*-value
From baseline to 6 weeks	AOS	1.48 ± 37.64 (-11.11, 8.16)	-21.15 ± 23.59 (-26.61, -15.68)	< 0.01*
	VAS	-1.69 ± 1.16 (-1.99, -1.39)	-1.26 ± 1.00 (-1.49, -1.03)	0.023*
	SF-36	12.41 ± 14.74 (8.63, 16.18)	10.67 ± 9.97 (8.35, 12.98)	0.416
From baseline to 12 weeks	AOS	0.61 ± 35.23 (-8.42, 9.63)	-20.74 ± 27.89 (-27.20, -14.28)	< 0.01*
	VAS	-1.84 ± 1.42 (-2.20, -1.47)	-1.92 ± 1.33 (-2.29, -1.61)	0.731
	SF-36	9.86 ± 18.31 (5.17, 14.57)	13.23 ± 13.04 (10.21, 16.25)	0.215

CI, confidence interval; AOS, ankle Osteoarthritis Scale; VAS, visual analogue scale; SF-36, short form-36

*p* values of < 0.05 were considered significant (marked as asterisk, “*”)

### Secondary outcomes

**Fig. 2:**
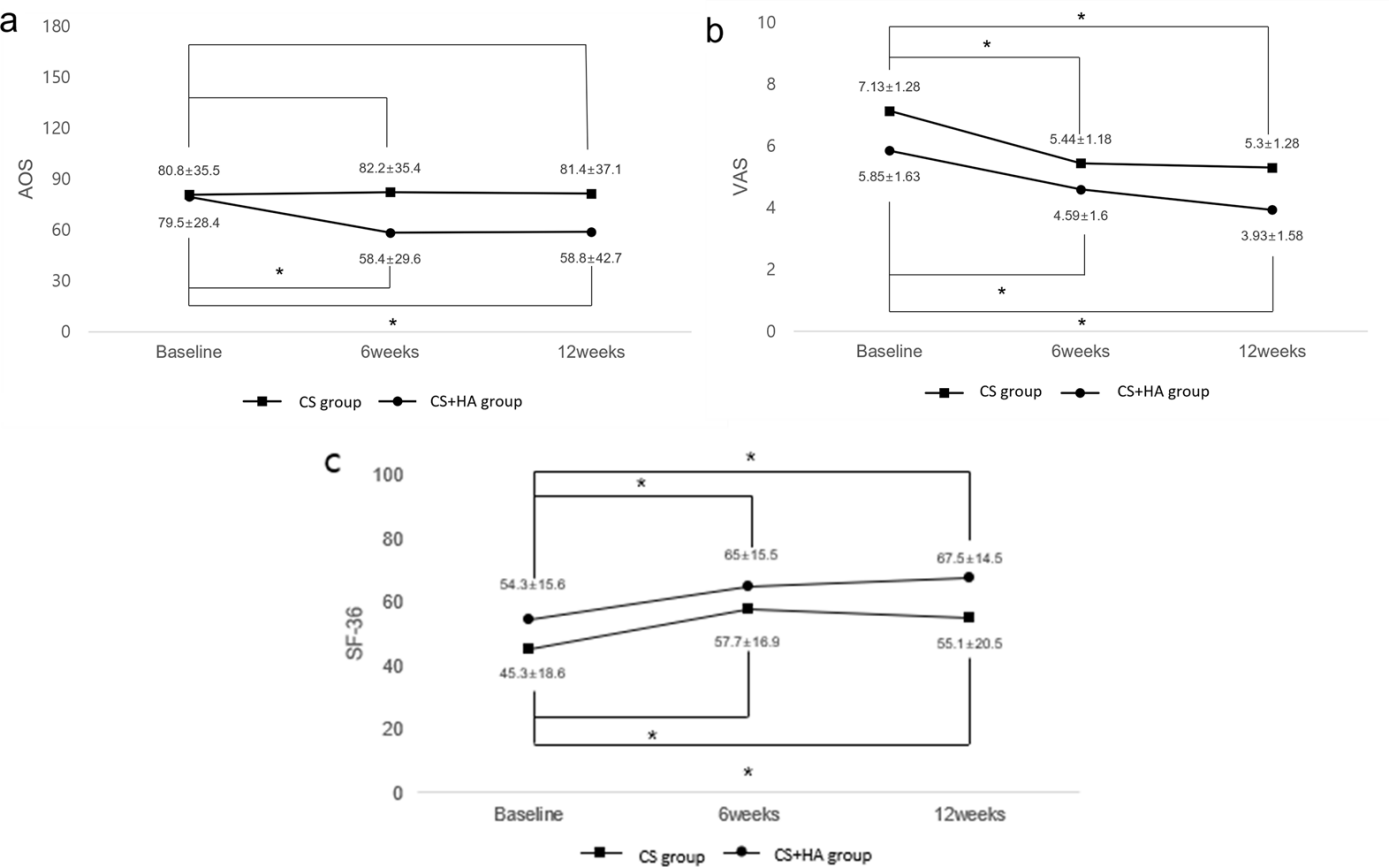
Clinical results including Ankle Osteoarthritis Scale (**A**), Visual Analogue Scale (**B**), Short Form-36 (**C**) between the groups. Results are presented as mean values ± standard deviations at each time point * Values followed by an asterisk denote significant differences (adjusted *p-*value according to post-hoc test was used, < 0.016*)*

Both groups exhibited significant improvements in VAS and SF-36 scores at 6 (*p* ≤ 0.01 for both groups) and 12 weeks (*p* ≤ 0.01 for both groups) after injection compared to baseline. When comparing the groups, the CS group showed a greater mean VAS improvement at 6 weeks (*p* = 0.023) but not at 12 weeks (*p* = 0.731). Both groups showed significant improvements in PCS scores of the SF-36 at 6 (*p* ≤ 0.01 for both groups) and 12 weeks (*p* ≤ 0.01 for both groups) after injection compared to baseline, but there were no significant differences between the groups at either 6 (*p* = 0.416) or 12 weeks (*p* = 0.215). No major or minor complications were reported in either group at 6 or 12 weeks after injection. The following results were described in detail in Table 3; Fig. 2a. and 2b.

### Correlation with modified Takakura classification

**Fig. 3:**
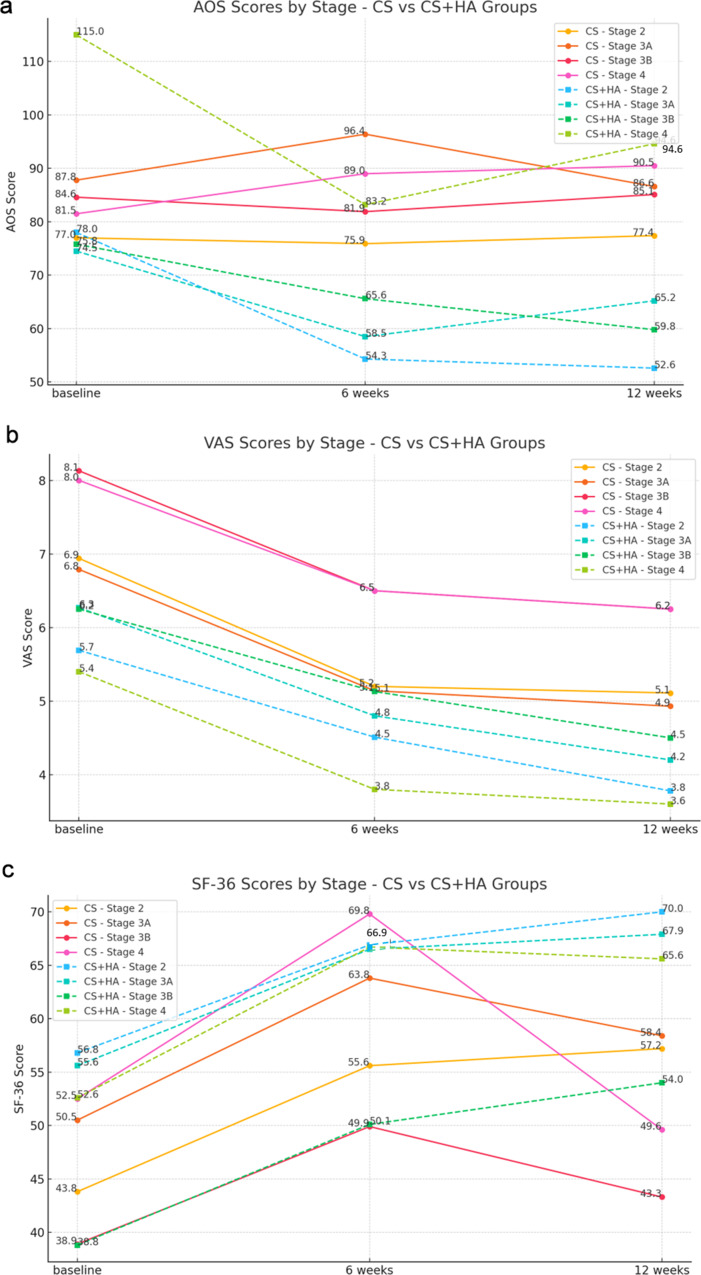
Illustrations of the changes in clinical outcomes (AOS, VAS, and SF-36) by group and time point across different OA stages are presented. Solid lines represent the CS group, while dotted lines represent the CS + HA group, showing the overall trend. All changes are depicted as differences from baseline

Figure 3 illustrates the changes in AOS, VAS, and SF-36 from baseline to 6 and 12 weeks after injection for two treatment groups across different stages of ankle OA. For AOS, the CS group shows minimal changes over time, whereas the CS + HA group exhibits a significant decrease, especially at stages 2 and 4. In VAS, both groups show a reduction over time, with the CS + HA group experiencing a more significant decline. The SF-36 scores increase for both groups, indicating an improvement. Overall, the CS + HA group demonstrates a tendency to exhibit more significant changes in comparison to the CS group across all parameters.

### MCID of each clinical parameter

The calculated MCID values for AOS, VAS, and SF-36 at each time point are shown in Table 4. Approximately half of the patients in both groups did not achieve the MCID for AOS (39% in the CS group and 49% in the CS + HA group at 6 weeks; 31% in the CS group and 39% in the CS + HA group at 12 weeks). For VAS, over 70% of patients in both groups achieved the MCID (76% in the CS group and 80% in the CS + HA group at 6 weeks; 76% in the CS group and 72% in the CS + HA group at 12 weeks). For SF-36, 48% in the CS group and 47% in the CS + HA group achieved the MCID at 6 weeks, and 47% in the CS group and 58% in the CS + HA group at 12 weeks.

**Table 4:** Calculated MCID values based on SD approach

		AOS	VAS	SF-36
CS	6 weeks after injection	18.82	0.58	7.37
	12 weeks after injection	17.11	0.71	4.96
CS + HA	6 weeks after injection	11.76	0.50	9.16
	12 weeks after injection	13.95	0.67	6.52

MCID, minimum clinically important difference; SD, standard deviation; AOS, ankle Osteoarthritis Scale; VAS, visual analogue scale; SF-36, short form-36

## Discussion

This randomized controlled trial compared the efficacy of dual intra-articular injections of CS + HA versus a single injection of CS in patients with ankle OA. The primary outcome, AOS scores, showed significantly greater improvement in the CS + HA group compared to the CS group at both 6 and 12 weeks. Secondary outcomes, including VAS and SF-36 scores, also showed significant improvements in both groups, with the CS + HA group generally exhibiting more substantial and sustained benefits, particularly in early-stage OA. The results suggest that dual injections provide superior and longer-lasting pain relief and functional improvement compared to corticosteroid alone, making it a more effective treatment option for ankle OA.

The results of our study are consistent with previous researches [27, 45, 46]. A meta-analysis of eight randomized controlled trials found that combined intra-articular injection of CS and HA resulted in greater short-term (2–4 weeks) and long-term (24–26 or maximum 52 weeks) reductions in WOMAC pain scale scores compared with HA alone for knee OA [27]. Another study showed significant pain relief with dual injections of HA and CS compared to a single CS injection for post-traumatic subtalar OA [47]. Despite some variations in results, the dual injection method was generally preferred. Notably, no previous studies have investigated this approach for the ankle OA.

Several conservative treatment options have been introduced for ankle OA, and an intra-articular injection is commonly indicated for patients showing inadequate response to medication [48]. Corticosteroid is one of the most commonly used agent because of their rapid onset of action and cost-effectiveness [49, 50]. However, recent studies have reported that its effects last only up to 4 weeks and that its adverse effects include cartilage and soft tissue destruction and cytotoxic effects on chondrocytes. Furthermore, infections, calcifications, and acute synovitis have been reported. Consequently, the execution of multiple corticosteroid injection poses challenges.

HA is a component of normal synovial fluid, in which it acts as a viscosity enhancer and volume expander, thus contributing to shock absorption [51]. In terms of pharmacokinetics, intra-articular HA remains effective for varying periods, depending on the molecular weight of the formulation and types of joints treated. The clinical effects of HA tend to act from days to several weeks. Kim et al. [24] reported HA products with an average molecular weights of ≥ 300 kDa have been effective in reducing pain at 12 weeks after the last injection for knee OA. Accordingly, the relevant product item and follow-up period was determined. Furthermore, Kotz et al. [52] reported in a multicenter study that the effects of intra-articular HA were maintained up to 12 months after injection in 55% of 108 patients with knee OA. Although various studies have reported varying durations for the effects of HA, our study adopted the 12-week follow-up period, which might be considered an enough period that can distinguish a shorter duration of CS monotherapy and long-term effects of dual therapy, given the short-term effects of CS monotherapy. 12-week period was considered as a common timeframe suggested by various studies [24, 53, 54]. In our study, we also verified the benefits of HA or HA + CS injection regimen for ankle OA and demonstrated treatment safety, optimal dosage [20, 55].

It is acknowledged that the difference in the number of injections between the two groups, which could be perceived as a limitation in maintaining the parallel design of this study. However, this imbalance was deliberately chosen based on some studies supporting the efficacy of multiple HA injections. A systemic review and meta-analysis by Concoff et al. [22] found that three weekly injections of HA provided significantly greater pain relief and functional improvement than a single injection. In addition, Witteveen et al. [23] reported that three weekly injections of HA resulted in better clinical outcomes than a single injection in patients with OA. Although this resulted in a different number of injections between two groups, the authors chose to provide an effective treatment regimen that reflects clinical practice and increase the potential benefits.

A low incidence of side effects has been reported with intra-articular HA injection [56]. Special caution is required for acute pseudo-septic arthritis or a synovial flare [57]. Its clinical presentation is similar to infectious arthritis, but its mechanism is known to be related to high production of proinflammatory cytokines and hypersensitivity reactions; hyaluronan and CD44 have a ligand-receptor association that may increase the recruitment of inflammatory cells [58]. Because it is not a self-limiting disease, anti-inflammatory treatment is necessary in many cases. Once septic arthritis is excluded, intra-articular corticosteroid could be one of the treatment options [57, 59, 60]. On the other hand, the possible adverse effect related to CS includes cartilage deterioration, especially with repeated uses. One relevant study has shown that CS may lead to accelerated cartilage breakdown with frequent injections [53]. In order to prevent its effect, in this study, single usage of CS is determined. There have been no documented side effects specific to the combination use of CS and HA in previous studies. Therefore, special cautions were prepared to address the side effects commonly associated with each individual injection. Fortunately, none of the major adverse effects previously mentioned were reported throughout this study.

Several studies on HA injections have indicated a link between OA stages and treatment effectiveness. One such study found that early- and mid- stage knee OA (Kellgren-Lawrence stage 2 or 3) exhibited greater clinical improvements compared to end-stage OA [61, 62]. In a prospective study by Lee et al. [19], 37 patients with ankle OA received three weekly HA injections. The study compared baseline AOS, VAS, and AOFAS ankle-hindfoot scores with those at 6 months after injection across different OA stages. The results suggested that clinical outcomes worsened with advancing stages, with HA injections reducing pain more effectively in early or intermediate-grade ankle OA. Our study found similar results: patients in the CS + HA groups with early-stage OA (Stage 2 and 3 A) showed significant clinical improvements, while those with end-stage OA (Stage 3B and 4) did not. Thus, we believe that CS provides effective short-term pain relief, while HA offered longer-term benefits.

In the present study, MCID values provide a critical benchmark for assessing the clinical significance of treatment outcomes. MCID values were calculated using both anchor-based and distribution-based methods. These values indicate that a significant proportion of patients experienced clinically meaningful improvements following the dual intra-articular injection, particularly in terms of pain relief and quality of life. These results are consistent with previous research indicating that achieving the MCID reflects meaningful clinical improvements [40, 41].

Our study has several limitations. First, although this was a randomized controlled trial, the difference in the number of injections between the two groups is a limitation. The CS group received one injection while the CS + HA group received three, which affects the blinding process. However, since the participants were not informed of the regimen and number of injections, we consider this study to be single-blind. In addition, the use of three HA injections as a control could address this limitation. Second, the relatively short follow-up period limits our ability to comment on the long-term effects of HA. One literature [49] suggests that the effects of HA begin to manifest 1 week after injection and peak between 5 and 13 weeks. Given the challenges of long-term follow-up for nonsurgical treatments, we chose 6- and 12-week follow-up visits to assess effectiveness. The reason we did not set a follow-up period longer than 12 weeks is that we determined CS would not be effective beyond this point. Therefore, we believed that this period would show the long-term effects we aimed. However, further studies with longer follow-up period over 12 weeks would strengthen our conclusions. Third, baseline scores before injection differed between the two groups. To address this, we compared median changes between pre- and post-treatment status rather than absolute values. Lastly, the discrepancy in the final number of patients enrolled (61 in the CS group versus 74 in the CS + HA group) was due to factors such as patient dropout and exclusion based on eligibility after randomization, not a flaw in the study design. Despite these differences, we maintained rigorous randomization and blinding protocols to minimize bias. We believe that further studies with longer follow-up periods and larger sample sizes are recommended to further validate these findings and explore the long-term effects of this treatment regimen.

## Conclusion

This randomized controlled trial demonstrates that dual intra-articular injections of corticosteroid and hyaluronic acid (CS + HA) provide superior pain relief and functional improvement compared with a single corticosteroid (CS) injection in patients with ankle osteoarthritis. Despite the limitations, our study supports the efficacy and safety of dual injections as a more effective treatment option for ankle OA.

## Electronic supplementary material

Below is the link to the electronic supplementary material.

Supplementary Material 1

Supplementary Material 2

## Data Availability

The datasets used and/or analysed during the current study are available from the corresponding author on reasonable request.

## Declarations

The study adheres to CONSORT guidelines.

## Abbreviations

CS: Corticosteroid
HA: Hyaluronic acid
TA: Tibialis anterior
OA: Osteoarthritis
AOS: Ankle osteoarthritis scale
VAS: Visual analog scale
SF-36: Short form of 36
PCS: Physical component summary
ANOVA: Analysis of variance
MCID: Minimum clinically important differences

## References

[1] Tejero S, Prada-Chamorro E, González-Martín D, García-Guirao A, Galhoum A, Valderrabano V, Herrera-Pérez M. Conservative treatment of ankle osteoarthritis. J Clin Med. 2021;10(19):4561. 34640583 10.3390/jcm10194561 PMC8509213

[2] Coe MP, Sutherland JM, Penner MJ, Younger A, Wing KJ. Minimal clinically important difference and the effect of clinical variables on the ankle osteoarthritis scale in surgically treated end-stage ankle arthritis. J Bone Joint Surg Am. 2015;97(10):818–23. 25995492 10.2106/JBJS.N.00147

[3] Schmid T, Krause FG. Conservative treatment of asymmetric ankle osteoarthritis. Foot Ankle Clin. 2013;18(3):437–48. 24008210 10.1016/j.fcl.2013.06.003

[4] Jantzen C, Ebskov LB, Andersen KH, Benyahia M, Rasmussen PB, Johansen JK. The effect of a single hyaluronic acid injection in ankle arthritis: A prospective cohort study. J Foot Ankle Surg. 2020;59(5):961–3. 32475656 10.1053/j.jfas.2020.03.015

[5] Ward ST, Williams PL, Purkayastha S. Intra-articular corticosteroid injections in the foot and ankle: a prospective 1-year follow-up investigation. J Foot Ankle Surg. 2008;47(2):138–44. 18312921 10.1053/j.jfas.2007.12.007

[6] Grice J, Marsland D, Smith G, Calder J. Efficacy of foot and ankle corticosteroid injections. Foot Ankle Int. 2016;38(1):8–13. 27672014 10.1177/1071100716670160

[7] MacMahon PJ, Eustace SJ, Kavanagh EC. Injectable corticosteroid and local anesthetic preparations: A review for radiologists. Radiology. 2009;252(3):647–61. 19717750 10.1148/radiol.2523081929

[8] Cole BJ, Schumacher RH Jr. Injectable corticosteroids in modern practice. JAAOS - J Am Acad Orthop Surg. 2005, 13(1). 10.5435/00124635-200501000-00006 15712981

[9] Rhon DI, Kim M, Asche CV, Allison SC, Allen CS, Deyle GD. Cost-effectiveness of physical therapy vs Intra-articular glucocorticoid injection for knee osteoarthritis: A secondary analysis from a randomized clinical trial. JAMA Netw Open. 2022;5(1):e2142709–2142709. 35072722 10.1001/jamanetworkopen.2021.42709 PMC8787617

[10] Askari A, Gholami T, NaghiZadeh MM, Farjam M, Kouhpayeh SA, Shahabfard Z. Hyaluronic acid compared with corticosteroid injections for the treatment of osteoarthritis of the knee: a randomized control trail. Springerplus. 2016;5:442. 27104130 10.1186/s40064-016-2020-0 PMC4828353

[11] Raynauld JP, Buckland-Wright C, Ward R, Choquette D, Haraoui B, Martel-Pelletier J, Uthman I, Khy V, Tremblay JL, Bertrand C, et al. Safety and efficacy of long-term intraarticular steroid injections in osteoarthritis of the knee: a randomized, double-blind, placebo-controlled trial. Arthritis Rheum. 2003;48(2):370–7. 12571845 10.1002/art.10777

[12] Onoi Y, Hiranaka T, Hida Y, Fujishiro T, Okamoto K, Matsumoto T, Kuroda R. Second-Look arthroscopic findings and clinical outcomes after Adipose-Derived regenerative cell injection in knee osteoarthritis. Clin Orthop Surg. 2022;14(3):377–85. 36061847 10.4055/cios20312 PMC9393284

[13] Sun SF, Hsu CW, Sun HP, Chou YJ, Li HJ, Wang JL. The effect of three weekly intra-articular injections of hyaluronate on pain, function, and balance in patients with unilateral ankle arthritis. J Bone Joint Surg Am. 2011;93(18):1720–6. 21938376 10.2106/JBJS.J.00315

[14] Chang KV, Hsiao MY, Chen WS, Wang TG, Chien KL. Effectiveness of intra-articular hyaluronic acid for ankle osteoarthritis treatment: a systematic review and meta-analysis. Arch Phys Med Rehabil. 2013;94(5):951–60. 23149311 10.1016/j.apmr.2012.10.030

[15] Tran K, Loshak H. CADTH Rapid Response Reports. In: Intra-Articular Hyaluronic Acid for Viscosupplementation in Osteoarthritis of the Hand, Shoulder, and Temporomandibular Joint: A Review of Clinical Effectiveness and Safety. edn. Ottawa (ON): Canadian Agency for Drugs and Technologies in Health Copyright © 2019 Canadian Agency for Drugs and Technologies in Health. 2019. 31553549

[16] He WW, Kuang MJ, Zhao J, Sun L, Lu B, Wang Y, Ma JX, Ma XL. Efficacy and safety of intraarticular hyaluronic acid and corticosteroid for knee osteoarthritis: A meta-analysis. Int J Surg. 2017;39:95–103. 28137554 10.1016/j.ijsu.2017.01.087

[17] Park CH, Park JJ, Seok HG, Woo IH. The efficacy of Intra-Articular hyaluronic acid injections in ankle osteoarthritis. JKFAS. 2022;26(2):71–7.

[18] Creamer P, Hochberg MC. Osteoarthritis. Lancet. 1997;350(9076):503–8. 9274595 10.1016/S0140-6736(97)07226-7

[19] Lee GW, Kwak WK, Lee KB. Effects and safety of Intra-Articular sodium hyaluronate injection for the treatment of ankle osteoarthritis: A prospective clinical trial. J Foot Ankle Surg. 2022;61(2):345–9. 34801379 10.1053/j.jfas.2021.09.012

[20] Salk RS, Chang TJ, D’Costa WF, Soomekh DJ, Grogan KA. Sodium hyaluronate in the treatment of osteoarthritis of the ankle: a controlled, randomized, double-blind pilot study. J Bone Joint Surg Am. 2006;88(2):295–302. 16452740 10.2106/JBJS.E.00193

[21] Sun S-F, Chou Y-J, Hsu C-W, Chen W-L. Hyaluronic acid as a treatment for ankle osteoarthritis. Curr Rev Musculoskelet Med. 2009;2(2):78–82. 19468874 10.1007/s12178-009-9048-5 PMC2697335

[22] Concoff A, Sancheti P, Niazi F, Shaw P, Rosen J. The efficacy of multiple versus single hyaluronic acid injections: a systematic review and meta-analysis. BMC Musculoskelet Disord. 2017;18(1):542. 29268731 10.1186/s12891-017-1897-2 PMC5740709

[23] Witteveen AG, Sierevelt IN, Blankevoort L, Kerkhoffs GM, van Dijk CN. Intra-articular sodium hyaluronate injections in the Osteoarthritic ankle joint: effects, safety and dose dependency. Foot Ankle Surg. 2010;16(4):159–63. 21047602 10.1016/j.fas.2009.10.003

[24] Kim JG, Kim K-I, Park K-B, Park Y-G, Bae JH, Seo Y-J, Seon J-K, Shon OJ, Ahn JH, Wang L, et al. Safety and effectiveness of intra-articular injection of a highly cross-linked hyaluronic acid, LBSA0103 (Synovian): results from a post-marketing surveillance study in South Korea. PLoS ONE. 2023;18(6):e0287222. 37347765 10.1371/journal.pone.0287222 PMC10287010

[25] Paskins Z, Hughes G, Myers H, Hughes E, Hennings S, Cherrington A, Evans A, Holden M, Stevenson K, Menon A, et al. A randomised controlled trial of the clinical and cost-effectiveness of ultrasound-guided intra-articular corticosteroid and local anaesthetic injections: the hip injection trial (HIT) protocol. BMC Musculoskelet Disord. 2018;19(1):218. 30021588 10.1186/s12891-018-2153-0 PMC6052622

[26] Saltzman BM, Leroux T, Meyer MA, Basques BA, Chahal J, Bach BR, Yanke AB, Cole BJ. The therapeutic effect of Intra-articular normal saline injections for knee osteoarthritis: A Meta-analysis of evidence level 1 studies. Am J Sports Med. 2016;45(11):2647–53. 28027657 10.1177/0363546516680607

[27] Smith C, Patel R, Vannabouathong C, Sales B, Rabinovich A, McCormack R, Belzile EL, Bhandari M. Combined intra-articular injection of corticosteroid and hyaluronic acid reduces pain compared to hyaluronic acid alone in the treatment of knee osteoarthritis. Knee Surg Sports Traumatol Arthrosc. 2019;27(6):1974–83. 30046992 10.1007/s00167-018-5071-7

[28] Tanaka Y, Takakura Y, Hayashi K, Taniguchi A, Kumai T, Sugimoto K. Low tibial osteotomy for varus-type osteoarthritis of the ankle. J Bone Joint Surg Br. 2006;88(7):909–13. 16798994 10.1302/0301-620X.88B7.17325

[29] Kim J-B, Park CH, Ahn J-Y, Kim J, Lee W-C. Characteristics of medial gutter arthritis on weightbearing CT and plain radiograph. Skeletal Radiol. 2021;50(8):1575–83. 33410964 10.1007/s00256-020-03688-2

[30] Park CH, Kim JB, Kim J, Yi Y, Lee W-C. Joint preservation surgery for Varus ankle arthritis with large Talar Tilt. Foot Ankle Int. 2021;42(12):1554–64. 34315278 10.1177/10711007211027290

[31] Hamawandi SA. Use of hyaluronic acid injection after arthroscopic release in lateral patellar compression syndrome with degenerative cartilage changes: randomized control trial. BMC Musculoskelet Disord. 2021;22(1):24. 33407337 10.1186/s12891-020-03876-0 PMC7786499

[32] de Cesar Netto C, da Fonseca LF, Simeone Nascimento F, O’Daley AE, Tan EW, Dein EJ, Godoy-Santos AL, Schon LC. ☆Diagnostic and therapeutic injections of the foot and ankle—An overview. Foot Ankle Surg. 2018;24(2):99–106. 29409219 10.1016/j.fas.2017.02.001

[33] Domsic RT, Saltzman CL. Ankle osteoarthritis scale. Foot Ankle Int. 1998;19(7):466–71. 9694125 10.1177/107110079801900708

[34] Martin DP, Engelberg R, Agel J, Swiontkowski MF. Comparison of the musculoskeletal function assessment questionnaire with the short form-36, The Western Ontario and McMaster Universities Osteoarthritis Index, and the sickness impact profile health-status measures*. *JBJS* 1997; 79(9). 10.2106/00004623-199709000-00006 9314394

[35] Sun S-F, Chou Y-J, Hsu C-W, Hwang C-W, Hsu P-T, Wang J-L, Hsu Y-W, Chou M-C. Efficacy of intra-articular hyaluronic acid in patients with osteoarthritis of the ankle: a prospective study. Osteoarthr Cartil. 2006;14(9):867–74. 10.1016/j.joca.2006.03.003 16635582

[36] Mathai SC, Puhan MA, Lam D, Wise RA. The minimal important difference in the 6-Minute walk test for patients with pulmonary arterial hypertension. Am J Respir Crit Care Med. 2012;186(5):428–33. 22723290 10.1164/rccm.201203-0480OC PMC3443803

[37] Gandek B, Sinclair SJ, Kosinski M, Ware JE Jr. Psychometric evaluation of the SF-36 health survey in medicare managed care. Health Care Financ Rev. 2004;25(4):5–25. 15493441 PMC4194895

[38] Faul F, Erdfelder E, Buchner A, Lang A-G. Statistical power analyses using G*Power 3.1: tests for correlation and regression analyses. Behav Res Methods. 2009;41(4):1149–60. 19897823 10.3758/BRM.41.4.1149

[39] Nahm FS. Understanding effect sizes. Hmr. 2015;35(1):40–3.

[40] Sedaghat AR. Understanding the minimal clinically important difference (MCID) of Patient-Reported outcome measures. Otolaryngol Head Neck Surg. 2019;161(4):551–60. 31159641 10.1177/0194599819852604

[41] Franceschini M, Boffa A, Pignotti E, Andriolo L, Zaffagnini S, Filardo G. The minimal clinically important difference changes greatly based on the different calculation methods. Am J Sports Med. 2023;51(4):1067–73. 36811558 10.1177/03635465231152484 PMC10026158

[42] Chen C, Li Z, Zhang Y, Zhou H, Li Y, He W, Ye T, Yang Y. What’s the clinical significance of VAS, AOFAS, and SF-36 in progressive collapsing foot deformity. Foot Ankle Surg. 2024;30(2):103–9. 37858492 10.1016/j.fas.2023.10.002

[43] Sutherland JM, Albanese CM, Wing K, Zhang YJ, Younger A, Veljkovic A, Penner M. Effect of patient demographics on minimally important difference of ankle osteoarthritis scale among End-Stage ankle arthritis patients. Foot Ankle Int. 2021;42(5):624–32. 33504200 10.1177/1071100720977842 PMC8127667

[44] Sutton RM, McDonald EL, Shakked RJ, Fuchs D, Raikin SM. Determination of minimum clinically important difference (MCID) in visual analog scale (VAS) pain and foot and ankle ability measure (FAAM) scores after hallux Valgus surgery. Foot Ankle Int. 2019;40(6):687–93. 30841749 10.1177/1071100719834539

[45] Oh SH, Sung WS, Oh SH, Jo CH. Comparative analysis of intra-articular injection of steroid and/or sodium hyaluronate in adhesive capsulitis: prospective, double-blind, randomized, placebo-controlled study. JSES Int. 2021;5(6):1091–104. 34766090 10.1016/j.jseint.2021.07.017 PMC8568987

[46] Wang CP, Lee WC, Hsieh RL. Effects of repeated Co-Injections of corticosteroids and hyaluronic acid on knee osteoarthritis: A prospective, Double-Blind randomized controlled trial. Am J Med. 2022;135(5):641–9. 34958762 10.1016/j.amjmed.2021.11.016

[47] Gomes FF, Maranho DA, Gomes MS, de Castro IM, Mansur H. Effects of hyaluronic acid with Intra-articular corticosteroid injections in the management of subtalar Post-traumatic Osteoarthritis – Randomized comparative trial. J Foot Ankle Surg. 2023;62(1):14–20. 35752551 10.1053/j.jfas.2022.03.003

[48] Witteveen AG, Hofstad CJ, Kerkhoffs GM. Hyaluronic acid and other Conservative treatment options for osteoarthritis of the ankle. Cochrane Database Syst Rev. 2015;2015(10):Cd010643. 26475434 10.1002/14651858.CD010643.pub2 PMC9254328

[49] Jüni P, Hari R, Rutjes AW, Fischer R, Silletta MG, Reichenbach S, da Costa BR. Intra-articular corticosteroid for knee osteoarthritis. Cochrane Database Syst Rev. 2015;2015(10):Cd005328. 26490760 10.1002/14651858.CD005328.pub3 PMC8884338

[50] Mankin HJ, Conger KA. The acute effects of intra-articular hydrocortisone on articular cartilage in rabbits. J Bone Joint Surg Am. 1966;48(7):1383–8. 5921793

[51] Gupta RC, Lall R, Srivastava A, Sinha A. Hyaluronic acid: molecular mechanisms and therapeutic trajectory. Front Vet Sci. 2019;6:192. 31294035 10.3389/fvets.2019.00192 PMC6603175

[52] Kotz R, Kolarz G. Intra-articular hyaluronic acid: duration of effect and results of repeated treatment cycles. Am J Orthop (Belle Mead NJ). 1999;28(11 Suppl):5–7. 10587245

[53] Ayub S, Kaur J, Hui M, Espahbodi S, Hall M, Doherty M, Zhang W. Efficacy and safety of multiple intra-articular corticosteroid injections for osteoarthritis—a systematic review and meta-analysis of randomized controlled trials and observational studies. Rheumatology. 2021;60(4):1629–39. 33432345 10.1093/rheumatology/keaa808

[54] Rodriguez-Merchan EC. Intra-articular injections of hyaluronic acid and other drugs in the knee joint. HSS J ^®^. 2013;9(2):180–2. 24426865 10.1007/s11420-012-9320-x PMC3757486

[55] Mei-Dan O, Kish B, Shabat S, Masarawa S, Shteren A, Mann G, Nyska M. Treatment of osteoarthritis of the ankle by intra-articular injections of hyaluronic acid: a prospective study. J Am Podiatr Med Assoc. 2010;100(2):93–100. 20237359 10.7547/1000093

[56] Evanich JD, Evanich CJ, Wright MB, Rydlewicz JA. Efficacy of intraarticular hyaluronic acid injections in knee osteoarthritis. Clin Orthop Relat Res. 2001;390:173–81. 10.1097/00003086-200109000-00020 11550864

[57] Aydın M, Arıkan M, Toğral G, Varış O, Aydın G. Viscosupplementation of the knee: three cases of acute pseudoseptic arthritis with painful and irritating complications and a literature review. Eur J Rheumatol. 2017;4(1):59–62. 28293455 10.5152/eurjrheum.2016.15075 PMC5335889

[58] Kurosaka N, Takagi T, Koshino T. Effects of hyaluronate on CD44 expression of infiltrating cells in exudate of rat air pouch, induced by sensitization with lipopolysaccharide. J Rhuematol. 1999;26(10):2186–90. 10529137

[59] Bernardeau C, Bucki B, Lioté F. Acute arthritis after intra-articular hyaluronate injection: onset of effusions without crystal. Ann Rheum Dis. 2001;60(5):518–20. 11302877 10.1136/ard.60.5.518 PMC1753628

[60] Martens PB. Bilateral symmetric inflammatory reaction to Hylan G-F 20 injection. Arthritis Rheum. 2001;44(4):978–9. 11315938 10.1002/1529-0131(200104)44:4<978::AID-ANR156>3.0.CO;2-N

[61] Bowman S, Awad ME, Hamrick MW, Hunter M, Fulzele S. Recent advances in hyaluronic acid based therapy for osteoarthritis. Clin Transl Med. 2018;7(1):6. 29450666 10.1186/s40169-017-0180-3 PMC5814393

[62] Wang CT, Lin J, Chang CJ, Lin YT, Hou SM. Therapeutic effects of hyaluronic acid on osteoarthritis of the knee. A meta-analysis of randomized controlled trials. J Bone Joint Surg Am. 2004;86(3):538–45. 14996880 10.2106/00004623-200403000-00012

