# Supplementary material for: Dual intra-articular injections of corticosteroid and hyaluronic acid versus single corticosteroid injection for ankle osteoarthritis: a randomized comparative trial
Source: BMC Musculoskelet Disord. 2025 Mar 11;26:239. doi: 10.1186/s12891-025-08488-0 (PMC11895146; doi:10.1186/s12891-025-08488-0)
Supplement: Supplementary file 1 — Supplementary Material 1 [file 12891_2025_8488_MOESM1_ESM.docx]

**Study Protocol**

| **Title:** **Dual Intra-articular injections** **of corticosteroid and hyaluronic acid are more effective than corticosteroid alone for ankle osteoarthritis**  **Short title: Efficacy of Intra-articular Corticosteroid and hyaluronic acid injection for Ankle Osteoarthriti****s**  Inha Woo^1^, Jung-Min Lee^2^, Jo Seong Hyeon^1^, Jongyoon Baek^3^, Chul Hyun Park^4^  ^1^ Department of Orthopaedic surgery, Yeungnam University Medical Center, Daegu 42415, Republic or Korea  ^2^ Department of Biomedical Engineering, Inje University, Gimhae, 50834, Republic of Korea  ^3^ Department of Anesthesiology and Pain Medicine, Yeungnam University College of Medicine, Daegu 42415, Republic of Korea.  ^4^ Department of Orthopaedic Surgery, Yeungnam University College of Medicine, Daegu 42415, Republic of Korea.  **Chief Investor Information (*):**  Chul Hyun Park, M.D., Ph.D., Department of Orthopaedic Surgery, College of Medicine, Yeungnam University, 170 Hyeonchung-ro, Nam-gu, Daegu 42415, Republic of Korea.  E-mail: chpark77@naver.com  Tel : +82-53-620-3640  Fax: +82-53-628-4020  Mobile : +82-10-5511-1843  **1. Background** | |
| --- | --- |
| CRIS Registration Number | KCT0008690 |
| Unique Protocol ID | 2019-09-063 |
| Public/Brief Title | Dual Intra-articular injections of corticosteroid and hyaluronic acid are more effective than corticosteroid alone for ankle osteoarthritis  Short title: Efficacy of Intra-articular Corticosteroid and hyaluronic acid injection for Ankle Osteoarthritis |
| Scientific Title | A prospective randomized study on the effects of clinical use of hyaluronic acid in patients with ankle osteoarthritis lesions |
| Acronym | OA; osteoarthritis, CS; corticosteroid, HA; hyaluronic acid, SF-36; Short form - 36 |
| MFDS Regulated Study | No |
| IND/IDE Protocol | No |
| Registered at Other Registry | No |
| Healthcare Benefit Approval Status | Not applicable |
| Institutional Review Board Information | |
| **2. Institutional Review Board / Ethics Committee** | |
| Board Approval Status | Submitted approval |
| Board Approval Number | YUMC 2019-09-063-011 |
| Approval Date, First registration date | 2019-12-24 |
| Institutional Review Board Name | Institutional Review Board of Yeungnam University Medical Center |
| Institutional Review Board Address | 170, Hyeonchung-ro, Nam-gu, Daegu, Republic of Korea |
| Institutional Review Board Telephone | +82-053-624-8352 |

| **3. Contact Details** | | |
| --- | --- | --- |
| **Contact Person for Principal Investigator / Scientific Queries** | | |
| Name | Chul Hyun Park, MD, PhD | |
| Title | Professor | |
| Telephone | +82-53-620-3620 | |
| Affiliation | Yeungnam University Medical Center | |
| Address | Orthopaedic surgery room, West 609 room, Hyun Chung Lo 170 , Nam-gu, Daegu, Republic of Korea | |
| **Contact Person for Public Queries** | | |
| Name | Chul Hyun Park, MD, PhD | |
| Title | Professor | |
| Telephone | +82-53-620-3620 | |
| Affiliation | Yeungnam University Medical Center | |
| Address | Orthopaedic surgery room, West 609 room, Hyun Chung Lo 170, Nam-gu, Daegu | |
| **Contact Person for Updating Information** | | |
| Name | Chul Hyun Park, MD, PhD | |
| Title | Professor | |
| Telephone | +82-53-620-3620 | |
| Affiliation | Yeungnam University Medical Center | |
| Address | Orthopaedic surgery room, West 609 room, Hyun Chung Lo 170, Nam-gu, Daegu | |
| **4. Status** | |  |
| Study Site | Single |  |
| Overall Recruitment Status | Completed |  |
| Date of First Enrollment | 2019-12-24 Actual date |  |
| Target Number of Participant | 160 |  |
| Primary Completion Date | 2021-10-28 , Actual |  |
| Study Completion Date | 2023-05-31 , Actual |  |
| **Recruitment Status by Participating Study Site 1** | | |
| Name of Study | Yeungnam University Medical Center |  |
| Recruitment Status | Completed |  |
| Date of First Enrollment | 2019-12-24 , |  |

|  | |
| --- | --- |
| **5. Source of Monetary / Material Support** | |
| **1. Source of Monetary/Material Support** | |
| Organization Name | Yeungnam University Medical Center |
| Organization Type | Medical Institute |
| Project ID |  |
| **6. Sponsor Organization** | |
| **1. Sponsor Organization** | |
| Organization Name | Yeungnam University Medical Center |
| Organization Type | Medical Institute |

| **7. Study Summary** | | | |
| --- | --- | --- | --- |
| Lay Summary | | Ankle osteoarthritis treatment can be divided into surgical treatment or non-surgical treatment depending on the severity.  Non-surgical treatment includes drug treatment, and according to the guideline for the treatment of osteoarthritis published by OsteoArthritis Research Society International (OARSI) and The European League Against Rheumatism (EULAR), it is recommended to use simple pain relievers with few side effects first in drug treatment, and if pain is not controlled, it is recommended to use non-steroidal anti-inflammatory drugs (NSAIDs) at the lowest effective dose, and even taking these drugs is not effective. Intra-articular injections of corticosteroid or hyaluronate, or use of stronger analgesics such as opioids are recommended. Hyaluronic acid is a drug commonly used for pain control in the shoulder and knee joints. Among the drugs used for pain control in the ankle joint, the effectiveness of hyaluronic acid has not yet been verified, so research on this is thought to contribute to the conservative treatment of patients with ankle osteoarthritis.  In this regard, in order to evaluate the effect of hyaluronic acid on ankle arthritis, we prospectively collected about 160 patients with ankle arthritis who visited a high-end hospital to observe clinical scores and imaging changes. Through randomization protocol, all eligible patients are allocated into two groups: CS(corticosteroid) group, CS + HA(Hyaluronic acid) gorup | |
| **8. Study Design** | | | |
| Study Type | | | Interventional Study |
| Study Purpose | | | Treatment |
| Phase | | | Not applicable |
| Intervention Model | | | Parallel |
| Blinding/Masking | | | Single |
| Blinded Subject | | | Subject |
| Allocation | | | RCT (Randomized controlled trial) |
| Intervention Type | | | Drug |
| Intervention Description | | | Before first injections, radiographic evaluations were performed using weight-bearing ankle anteroposterior (AP) and lateral radiographs and hindfoot alignment radiographs. All radiographs were obtained digitally, and radiographic parameters were measured using a Picture Archiving Communication System (PACS; Infinity, Seoul, Korea). Ankle OA was classified using the modified Takakura classification. An orthopaedic attending professor (C.H.P) and an orthopaedic resident (I.H.W) independently determined classifications twice at four-week intervals independently. When disagreement arose, the patient’s radiograph was replaced with another considered more representative until consensus was achieved.  A standard sterile skin preparation technique was performed around the ankle joint, and intra-articular injection was performed medial to the tibialis anterior tendon in the same location used for the anteromedial portal during ankle joint arthroscopy. Since this study was undertaken to investigate the effects of HA plus corticosteroid versus corticosteroid alonec, we decided to add HA to the conventional intra-articular corticosteroid injection regimen. For patients in the CS+HA group, 2 ml of HA (sodium HA, molecular weight, 3000 kDa; 2 mL, Hyruan Plus®; LG Life Sciences, Iksan, Korea) and 3 ml of mixture including 1 ml of corticosteroid (2.5 mg/ml, Triam®, ShinPoong Pharmaceuticals, Seoul, Korea), 1 ml of 0.5% bupivacaine (bupivacaine HCl®, Hana Pharm, Seoul, Korea), and 1 ml of normal saline were injected on the first week, followed by single injections of 2 ml of HA on the second and third weeks. In the CS group, 3 ml of mixture including 1 ml of corticosteroid, 1 ml of 0.5% bupivacaine, and 1 ml of normal saline were injected slowly.  CS: Corticosteoroid (Triamcinolone)  HA: Hyaluronic acid |
| Number of Arms | | | 2 |
| Arm 1 | Arm Label | | Corticosteoid group (CS) |
|  | Target Number of Participant | | 80 |
|  | Arm Type | | Active comparator |
|  | Arm Description | | In the CS group, 3 ml of mixture including 1 ml of corticosteroid, 1 ml of 0.5% bupivacaine, and 1 ml of normal saline were injected slowly. |
| Arm 2 | Arm Label | | Corticosteorid plus hyaluronic acid group (HA + CS group) |
|  | Target Number of Participant | | 80 |
|  | Arm Type | | Experimental |
|  | Arm Description | | For patients in the CS+HA group, 2 ml of HA (sodium HA, molecular weight, 3000 kDa; 2 mL, Hyruan Plus®; LG Life Sciences, Iksan, Korea) and 3 ml of mixture including 1 ml of corticosteroid (2.5 mg/ml, Triam®, ShinPoong Pharmaceuticals, Seoul, Korea), 1 ml of 0.5% bupivacaine (bupivacaine HCl®, Hana Pharm, Seoul, Korea), and 1 ml of normal saline were injected on the first week, followed by single injections of 2 ml of HA on the second and third weeks. |

|  | | | |
| --- | --- | --- | --- |
| **9. Subject Eligibility** | | | |
| Condition(s)/Problem(s) | | * (M00-M99)Diseases of the musculoskeletal system and connective tissue     (M15.0)Primary generalized (osteo)arthrosis   Ankle, Osteoarthritis | |
| Rare Disease | | No | |
| Inclusion Criteria | Gender | Both | |
|  | Age | 19Year~No Limit | |
|  | Description | A person diagnosed with ankle arthritis  Adults aged 19 years or older who can express their opinions  Person who consented to this study | |
| Exclusion Criteria | | Those with existing musculoskeletal disorders  A person who cannot perform injection treatment or who refuses treatment  Those who are pregnant or breastfeeding | |
| Side Effects related to the intervention | | Patients were sufficiently informed of possible complications. For assessment purposes, complications were dichotomized as major or minor. Minor complications included injection site pain and superficial swelling manageable without special procedures, and major complications included neurovascular injury and deep ankle joint infection requiring special treatment. Complications were assessed and recorded at each follow-up visit. The discontinuation of the study protocol was set as the occurrence of major complications or voluntary subject’s desire. | |
| Sample size calculation | | A priori power analysis was used to determine an appropriate sample size. A two-tailed matched-pairs t-test with an effect size for the primary outcome measure of 0.25, a probability of alpha error (significance) of 0.95, and a required power (1 – beta error probability) of 0.80, a total sample size of 128 was required. As a result, 135 patients were included in the study. | |
| Randomization process | | Randomization was conducted using a computer-generated allocation program (nQquery Advisor PPS 6.01, Saugus, MA, USA) that assigned numbers in strict chronologic. Randomization was stratified by age and OA stage, as defined by the modified Takakura classification. Each study participant was allocated a unique randomized number. | |
| **10. Outcome Measure(s)** | | |  |
| Type of Primary Outcome | | Efficacy |  |
| **Primary Outcome(s) 1** | | |  |
| Outcome | | The Ankle Osteoarthritis Scale (AOS) |  |
| Timepoint | | Pre injection, 6 weeks and 12 weeks after injection |  |
| **Secondary Outcome(s) 1,2** | | | |
| Outcome | | Visual analog scale, SF-36 |  |
| Timepoint | | Pre injection, 6 weeks and 12 weeks after injection |  |
| **Secondary Outcome(s) 3** | | | |
| Outcome | | Side effects related injection |  |
| Timepoint | | Pre injection, 6 weeks and 12 weeks after injection |  |

| **11. Study Publication** | |
| --- | --- |
| Result Published | No |
| **12. Sharing of Study Data(Deidentified Individual-Patient Data, IPD)** | |
| Sharing Statement | No |

Appendix I. AOS^1^


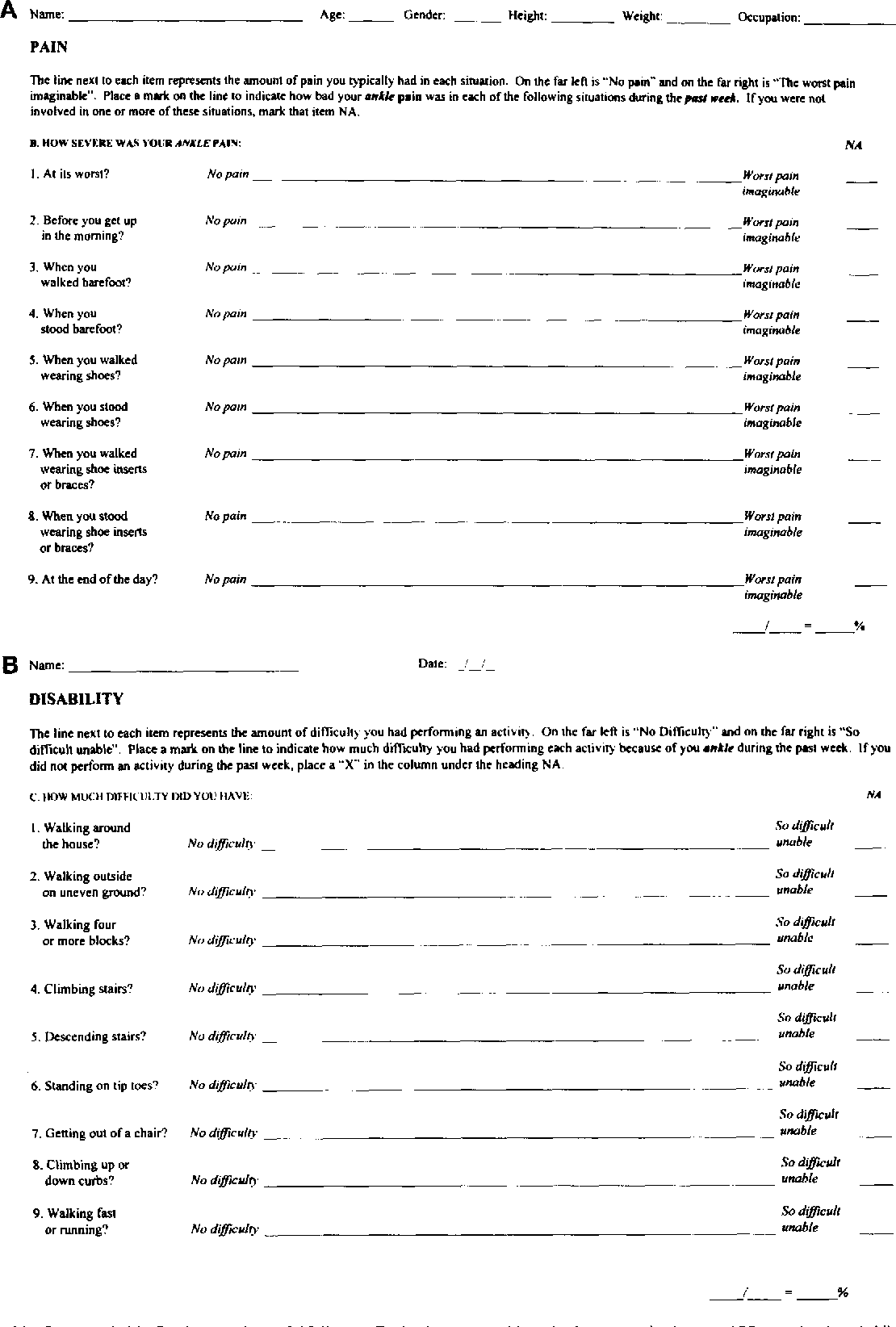


Appendix II. VAS


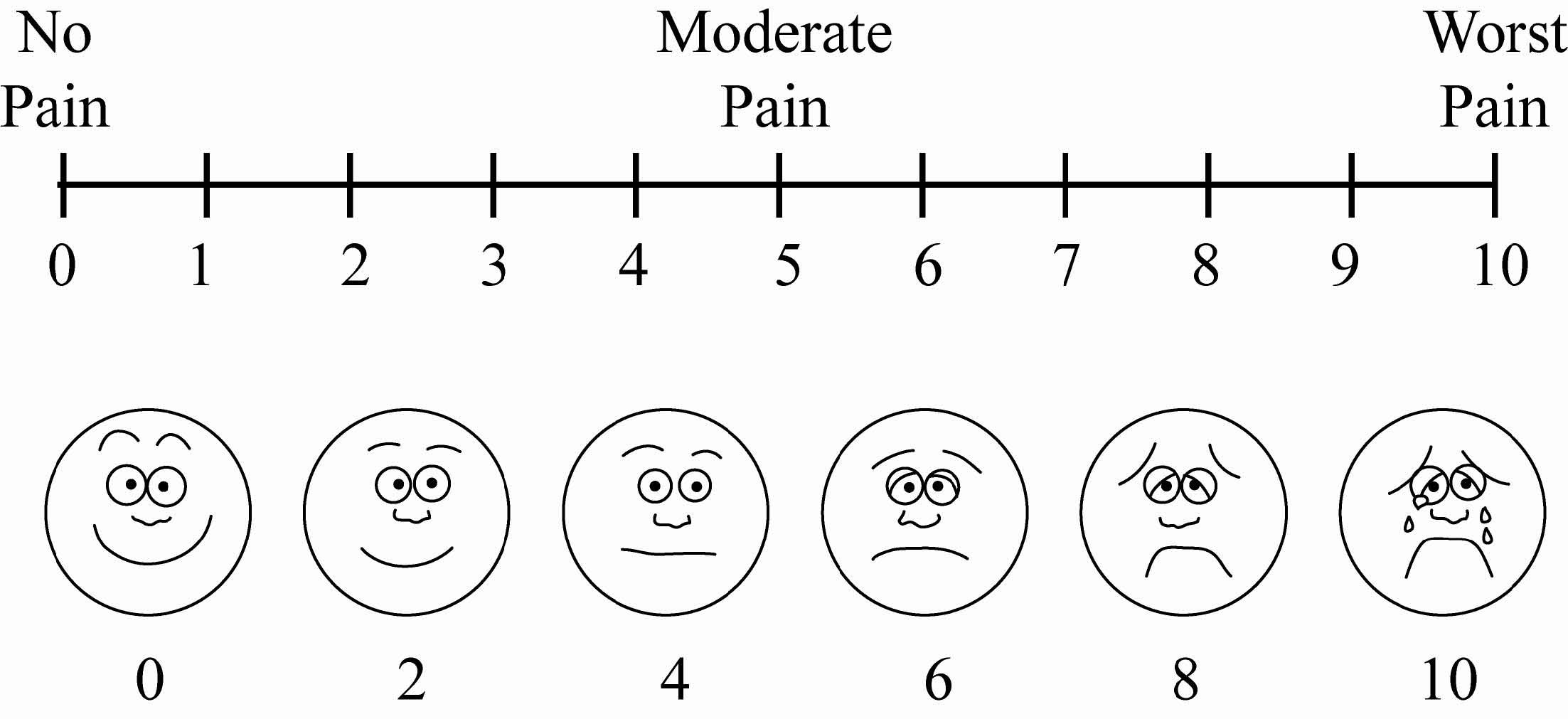


http://www.wikidoc.org/images/e/eb/Pain_scale.jpg

Appendix III. SF-36 survey^2^

**SF-36 Survey**

Date: / / Patient’s Name

**Visit: □ Pre-op**  **6 week □ 3 month**

**INSTRUCTIONS:** Please answer every question. Some questions may look like others, but each one is different. Please take the time to read and answer each question carefully by circling the number that best represents your response.

**1. In general, would you say your health is?**

| Excellent  (1) | Very Good (2) | Good  (3) | Fair (4) | Poor (5) |
| --- | --- | --- | --- | --- |

**2. Compared to one year ago, how would you rate your health in general now?**

| Much better now than one year ago  (1) | Somewhat better now than one year ago  (2) | About the same as one year ago  (3) | Somewhat worse now than one year ago  (4) | Much worse now than one year ago  (5) |
| --- | --- | --- | --- | --- |

**3. The following questions are about activities you might do during a typical day. Does your health now limit you in these activities? If so, how much:** (circle one number on each line)

|  | Yes, Limited A Lot | Yes, Limited A Little | No, Not Limited At All |
| --- | --- | --- | --- |
| A. **Vigorous activities**, such as running, lifting heavy objects participating in strenuous sports | 1 | 2 | 3 |
| B. **Moderate activities**, such as moving a table, pushing a vacuum cleaner, bowling, or playing golf | 1 | 2 | 3 |
| C. Lifting or carrying groceries | 1 | 2 | 3 |
| D. Climbing **several** flights of stairs | 1 | 2 | 3 |
| E. Climbing **one** flight of stairs | 1 | 2 | 3 |
| F. Bending, kneeling, or stooping | 1 | 2 | 3 |
| G. Walking **more than a mile** | 1 | 2 | 3 |
| H. Walking **several hundred yards** | 1 | 2 | 3 |
| I. Walking **one hundred yards** | 1 | 2 | 3 |
| J. Bathing or dressing yourself | 1 | 2 | 3 |

**4. During the past 4 weeks, how much of the time have you had any of the following problems with your work or other regular daily activities as a result of your physical health?** (Circle one number on each line)

|  | **All** the time | **Most** of the time | **Some** of the time | A **little** of the time | **None** of the time |
| --- | --- | --- | --- | --- | --- |
| A. Cut down on the **amount of time** you spend on work or other activities | 1 | 2 | 3 | 4 | 5 |
| B. **Accomplished less** than you would like | 1 | 2 | 3 | 4 | 5 |
| C. Were limited in the **kind** of work or other activities | 1 | 2 | 3 | 4 | 5 |
| D. Had **difficulty** performing the work or other activities (for example, it took extra effort) | 1 | 2 | 3 | 4 | 5 |

**5. During the past 4 weeks, how much of the time have you had any of the following problems with your work or other regular daily activities as a result of any emotional problems (such as feeling depressed or anxious)?** (Circle one number on each line)

|  | **All** the time | **Most** of the time | **Some** of the time | **A little** of the time | **None** of the time |
| --- | --- | --- | --- | --- | --- |
| A. Cut down on the **amount of time** you spend on work or other activities | 1 | 2 | 3 | 4 | 5 |
| B. **Accomplished less** than you would like | 1 | 2 | 3 | 4 | 5 |
| C. Did work or activities **less carefully than usual** | 1 | 2 | 3 | 4 | 5 |

**6. During the past 4 weeks, to what extent has your physical health or emotional problems interfered with your social activities with family, friends, neighbours, or groups?** (Circle one)

| Not at all  (1) | Slightly (2) | Moderately  (3) | Quite a bit (4) | Extremely  (5) |
| --- | --- | --- | --- | --- |

**7. How much bodily pain have you had during the past 4 weeks?** (Circle one)

| None  (1) | Very Mild (2) | Mild  (3) | Moderate  (4) | Severe (5) | Very Severe (6) |
| --- | --- | --- | --- | --- | --- |

**8. During the past 4 weeks, how much did pain interfere with your normal work (including both work outside the home and housework)?** (Circle one)

| Not at all  (1) | Slightly (2) | Moderately  (3) | Quite a bit (4) | Extremely  (5) |
| --- | --- | --- | --- | --- |

**9. These questions are about how you feel and how things have been with you during the past 4 weeks. For each question, please give the one answer that comes closest to the way you have been feeling. How much of the time during the past 4 weeks…** (Circle one number on each line)

|  | **All** the time | **Most** of the time | **Some** of the time | **A little** of the time | **None** of the time |
| --- | --- | --- | --- | --- | --- |
| A. did you feel full of life? | 1 | 2 | 3 | 4 | 5 |
| B. have you been very nervous? | 1 | 2 | 3 | 4 | 5 |
| C. have you felt so down in the dumps nothing could cheer you up? | 1 | 2 | 3 | 4 | 5 |
| D. have you felt calm and peaceful? | 1 | 2 | 3 | 4 | 5 |
| E. did you have a lot of energy? | 1 | 2 | 3 | 4 | 5 |
| F. have you felt downhearted and depressed? | 1 | 2 | 3 | 4 | 5 |
| G. did you feel worn out? | 1 | 2 | 3 | 4 | 5 |
| H. have you been happy? | 1 | 2 | 3 | 4 | 5 |
| I. did you feel tired? | 1 | 2 | 3 | 4 | 5 |

**10. During the past 4 weeks, how much of the time has your physical health or emotional problems interfered with your social activities (like visiting friends, relatives, etc.)?**

| All of the Time  (1) | Most of the Time (2) | Some of the Time  (3) | A Little of the Time (4) | None of the Time (5) |
| --- | --- | --- | --- | --- |

**11. How TRUE or FALSE is each of the following statements for you?** (Circle one number on each line)

|  | Definitely True | Mostly True | Don’t Know | Mostly False | Definitely False |
| --- | --- | --- | --- | --- | --- |
| A. I seem to get sick a little easier than other people | 1 | 2 | 3 | 4 | 5 |
| B. I am as healthy as anybody I know | 1 | 2 | 3 | 4 | 5 |
| C. I expect my health to get worse | 1 | 2 | 3 | 4 | 5 |
| D. My health is excellent | 1 | 2 | 3 | 4 | 5 |

**References**

1 Domsic, R. T. & Saltzman, C. L. Ankle osteoarthritis scale. *Foot Ankle Int* **19**, 466-471, doi:10.1177/107110079801900708 (1998).

2 McHorney, C. A., Ware, J. E., Jr., Lu, J. F. & Sherbourne, C. D. The MOS 36-item Short-Form Health Survey (SF-36): III. Tests of data quality, scaling assumptions, and reliability across diverse patient groups. *Med Care* **32**, 40-66, doi:10.1097/00005650-199401000-00004 (1994).
